# Perspective on the Genetic Response to Antiparasitics: A Review Article

**Published:** 2017

**Authors:** Patricia ALARCON-VALDES, Mariana ORTIZ-REYNOSO, Jonnathan SANTILLAN-BENITEZ

**Affiliations:** Faculty of Chemistry, Autonomous University of the State of Mexico (UAEM), Toluca, México

**Keywords:** Antiparasitics, Genetic response, Review article, Mexico

## Abstract

**Background:**

Drugs’ pharmacokinetics and pharmacodynamics can be affected by diverse genetic variations, within which simple nucleotide polymorphisms (SNPs) are the most common. Genetic variability is one of the factors that could explain questions like why a given drug does not have the desired effect or why do adverse drug reactions arise.

**Methods:**

In this retrospective observational study, literature search limits were set within PubMed database as well as the epidemiological bulletins published by the Mexican Ministry of Health, from Jan 1st 2001 to Mar 31st 2017 (16 years).

**Results:**

Metabolism of antiparasitic drugs and their interindividual responses are mainly modified by variations in cytochrome P450 enzymes. These enzymes show high frequencies of polymorphic variability thus affecting the expression of CYP2C, CYP2A, CYP2A6, CYP2D6, CYP2E6 and CYP2A6 isoforms. Research in this field opens the door to new personalized treatment approaches in medicine.

**Conclusion:**

Clinical and pharmacological utility yield by applying pharmacogenetics to antiparasitic treatments is not intended as a mean to improve the prescription process, but to select or exclude patients that could present adverse drug reactions as well as to evaluate genetic alterations which result in a diversity of responses, ultimately seeking to provide a more effective and safe treatment; therefore choosing a proper dose for the appropriate patient and the optimal treatment duration. Furthermore, pharmacogenetics assists in the development of vaccines. In other words, the aim of this discipline is to find therapeutic targets allowing personalized treatments.

## Introduction

Parasitic infections have historically had repercussions in the areas of health, society, and politics. These diseases are comprised of infections produced by protozoans, helminthes or ectoparasites. Their importance, based on the frequency, is geohelminthiasis, protozoan intestinal infection, malaria, schistosomiasis and lymphatic filariasis (1).

Probably, the parasitic infection having the most impact on human health, on a global scale, is malaria, for which an approximate annual mortality of 584000 people is reported, followed by protozoan intestinal infection, leishmaniasis and African trypanosomiasis (2–4).

## Methods

In this retrospective observational study, literature search limits were set within PubMed database as well as the epidemiological bulletins published by the Mexican Ministry of Health, from January 1st 2001 to March 31st 2017 (16 years).

## Results and Discussion

### Parasitic Intestinal Infections

Diseases related to intestinal parasites continue to be the most common cause of chronic endemic infections in developing countries found in tropical and subtropical areas, reason for which WHO has designated them as NTDs (Neglected Tropical Diseases) or non-attended tropical diseases. This classification is based on the following characteristics: geographical localization (tropical or subtropical, priority within health strategy plans, insufficient research, limited research funding and little intervention (5).

Due to a variety of eco-social factors such as ecological and climatic change, anthropogenic effects, such as human migration, urbanization, and industrialization, besides variation in host susceptibility towards parasites, together with genetic changes observed in parasites, as well as in their hosts. Infections caused by parasites present changes in their incidence and pathogenicity (6–8), insomuch that the prevalence of these diseases in the present day is not only rising in developing countries but also developed countries. To illustrate the aforementioned, in one of the reports generated by the Panamerican Health Organization (OPS) (9), in 14 countries in Latin America the prevalence of geohelminths is greater than 20% and in some cases it can reach 90%. These infections are considered by the WHO as a “generalized public health issue”.

One example that shows the rise in prevalence of parasitic infections in developed countries is that of two cohort studies done in France, where an increase in the presence of an intestinal protozoan, from 3% to 6.1% was observed (10); although not enough to reach the minimum threshold established by the WHO to be considered a public health issue (20%), this increase does reflect the need to review hygiene measures for water and food handling, as well as the migration of people from developing countries (11).

Despite the efforts to reduce the prevalence of parasitosis, there has been no significant change. There are more than 2 billion persons infected with at least one species of intestinal parasite and 4 billion having a risk of infection (12).

Human beings can be hosts to more than 72 genuses of protozoans and helminths, including the following: *Plasmodium, Trypanosoma, Leishmania, Giardia, Entamoeba, Blastocystis, Enterobius, Cistoisospora, Cyclospora, Cryptosporidium, Balantidium, Toxoplasma, Enterocytozoon, Encephalitozoon, Ascaris, Trichuris, Necator, Ancylostoma, Onchocerca, Hymenolepis, Taenia*, to name a few.

Several of these geniuses can cause intestinal parasitic infections, which continue to be a serious health issue, especially in countries with greater poverty; being the lead cause of parasitosis the contamination of food and water with fecal matter (13, 14).

### Intestinal parasites of medical importance and their classification

In this review, the traditional classification for parasites in humans is considered, adapted for its application to medical practice (15) (Table 1).

**Table 1:** Classification of the parasites which infect humans having major medical interest, adapted for its application to medical practice (15)

***Protozoans***	***Amoebas***	
	Flagellated	
	Sporozoos or Apicomplexa (coccidia)	
	Ciliates	
	Microsporidia (still considered to be fungi, for their genomics and proteomics)	
	“Others”, in which *Blastocystis* is found	
**Helminths**	Nematodes	
	Flatworms	Tapeworms
		Trematodes

### Impact of parasitosis on the health of the Mexican population

Mexico has a population of 119530753 inhabitants, according to the 2015 National census survey. Public health system reports epidemiological data indicating that more than 69% of people present intestinal parasitosis by pathogenic or commensal agents.

Among the causal agents most frequently associated, are the following: *Entamoeba histolytica, Giardia lamblia, and Blastocystis* spp. (protozoans); *Ascaris lumbricoides, Trichuris trichiura, Hymenolepis nana, Taenia saginata y Taenia solium* (helminths). Population studies report a frequency of 12% for parasitosis, identifying the presence of two or more intestinal parasites (16–18).

In Mexico, the national system of epidemiological surveillance (SINAVE) informs, through periodic bulletins, data on morbidity of diseases subject to epidemiological surveillance, in which intestinal parasitosis are found and considered a public health issue due to the magnitude of their incidence.

Likewise its transcendence is linked overall to secondary diseases such as anemia and surgical complications that occur in the cases of uncinariasis and ascariasis (18).

The broad geographical and socio-demographic diversity of Mexico allows for a high incidence in intestinal parasitosis among the general population According to the morbidity yearbook of 2015, an incidence rate of 279.28 per 100000 inhabitants corresponds to protozoan and 206.73 per 100000 inhabitants to helminth infections. This data evidences the importance of diagnosis, treatment, and prevention of intestinal parasitosis (19). Parasitosis affect physical growth and cognitive development in boys and girls (20).

### Antiparasitics used in Mexico

Antiparasitic treatment has evolved over the last century, from the use of antimonial inorganic compounds with activity against tissue protozoans such as *Leishmania*, to arsenic based preparations (i.e. Fowler’s arsenical liquor, an aqueous-alcoholic solution of sodium arsenite) as well as azoic colorants such as trypan blue; the two latter showing trypanocidal activity. Nitroimidazole ring molecules such as metronidazole (the drug of choice against protozoans) and heterocyclic nitrogenated compounds such as nitazoxanide have a broad antiparasitic spectrum (4, 17, 21, 22).

Currently, control of parasitic infections is mainly based on the use of drugs, because there are only a limited number of vaccines available against a few parasites (23).

The antiparasitic treatment applied in Mexico comprises drugs targeted against intestinal parasites, most commonly metronidazole, albendazole, mebendazole, nitazoxanide, tinidazole and secnidazole, for which an efficacy between 80% and 95% is reported. These drugs present adverse reactions such as abdominal pain, nausea, vomit and alterations of taste in cases where treatment duration is greater than 48 hours (4, 16–18).

Furthermore, for conventional regimes consisting of metronidazole, tinidazole and albendazole, treatment failures have been reported in approximately 20% of cases (24). The action of antiparasitics is based on its capacity of binding to certain sites of the parasitic structure and to inhibit important cellular functions (14). The majority of antiparasitic drugs usually have one of the following mechanisms of action (4):

- Affectation of biosynthetic metabolism: antiprotozoan drugs.
- Alteration of energetic metabolism, structural proteins or neuromuscular function: antihelminthic drugs.

**Fig. 1:**
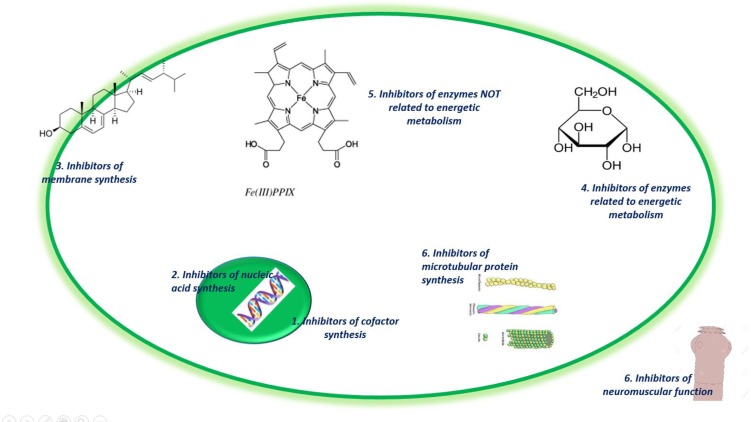
Pharmacological targets of main antiparasitic drugs of medical importance in humans (4)

The pharmacological targets of antiparasitics are mainly: receptors (nuclear, membrane or cytoplasmic), transport molecules, enzymes and ionic channels (25) (Fig. 1).

A brief of the inhibition mechanisms for proteins and structural molecules, as well as the biochemical mechanisms for selective action of the most representative antiparasitic drugs can be observed in Tables 2 and 3.

**Table 2:** Mechanism of inhibition of proteins and structural molecules (4, 21, 25)

***Inhibitors***	***Drug***	***Mechanism***	***Parasite***	***Example***
Of cofactor synthesis	Sulphonamides (sulphadoxine) Sulfones (dapsone)	Blockage of tetrahydrofolate biosynthesis, necessary in the synthesis of DNA (sulphadoxine, antagonist of paraaminobenzoic acid, is used to fix the dihydropteroate synthetase; it is administered with pyrimethamine; trimethoprim and proguanil inhibit dihydrofolate reductase	Sporozoos (coccidia )	*Plasmodium* spp.
	Diaminopyrimidines (trimethoprim, pyrimethamine) Proguanil			
Of the synthesis of nucleic acids	Chloroquines, Quinine	They insert themselves into the sequence of base pairs (Chloroquine inhibits polymerase)	Protozoans: (sporozoos-coccidia), anaerobic	*Trypanosoma cruzi* *Blastocystis* spp. *Giardia lamblia*
	Diamidines (pentamidine)	They intercalate between base pairs and act ionically		
	Benznidazole	Nitrogen group activation alkylates DNA		
	Nifurtimox			
	Metronidazole			
	Tinidazole			
Of membrane synthesis	Amphotericin B	Fixes itself to ergosterol, altering the permeability of the membrane, allowing the exit of K+ and other moleculas, in addition to oxidative process.	Protozoans: Tissue y free-living amoebas	*Leishmania* spp. *Naegleria* spp.
Of enzymes related to energetic metabolism	Melarsoprol (arsenicales trivalentes) Estibogluconato sódico Antimoniato de meglumina (antimoniales pentavalentes)	Blockage of kinases of glycolysis (pyruvate kinase)		*Trypanosoma* spp. *Leishmania* spp.
	Suramin	Inhibits glucose 6-phosphate dehydro-genase (G6PD) of the route of the pentoses		*Tryopanosoma* spp.
	Nitazoxanide	Inhibits pyruvate: ferredoxin óxido-reductase (PFOR)		*Cryptosporidium* spp. *Giardia lamblia*
	Primaquine Atovaquone	Blockage of mitocondrial transport of electrons, thus interfering with respiratory chain		*Plasmodium vuivax* *Plasmodium ovale* *Toxopolasma gondii*
Enzymes not related to energetic metabolism	Chloroquine	Inhibits hemopolymerase of the parasite and intoxicates it with iron, since it increases concentration of ferriprotoporphyin IX	Protozoans (sporozoos-coccidia)	*Plasmodium* spp.
	Eflornithine	Interferes in the biosynthesis of polyamines, irreversibly blocking la ornithine decarboxylase		*Trypanosoma* spp.
Of non-enzymatic proteins related to microtubular function	Carbamates Benzimidazoles (albendazole, mebendazole, triclabendazole)	Union to microtubules of the parasite, blocking assembly of tubulins. Alters secretion of acetylcholinesterase. Disminishes the incorporation of glucose to the parasite	Helminths	*Trichuris trichiura Áscaris lumbricoides*
Of the neuromuscular function	Levamisole	Interaction with the acetylcholine receptor (ACh), blocking the neuromuscular system of the parasite, increasing permeability in the membrane of the parasite, creating chlorine channels; ivermetic is also a GABA agonist		*Necator americanus*
	Pyrantel			*Ancylostoma duodenale*
	Metrifonate			*Enterobius vermicularis*
	Piperazine			*Wuchereria brancofti*
	Diethylcarbamazine			*Strongyloides stercolaris*
	Ivermectin			*Onchocerca volvulus*
	Praziquantel			*Toxocara* spp. *Fasciolopsis buski* *Hymenolepis* spp.

**Table 3:** Biochemical mechanisms for selective action of the main anti-protozoan drugs in humans (25)

***Antiparasitic drugs***	***Parasite***	***Biochemical mechanism***
Chloroquine	Protozoans (sporozoos-coccidia)	Different intake or secretion of the compound between the cell of the parasite and of the host
Pentamidine		
Metronidazole	Amoebas	Drug activates only within the parasite
	Ciliates: balantidiasis	
	Protozoans anaerobic: blastocystosis, giardiasis, trichomoniasis sporozoos (coccidia)	
Nifurtimox	Chagas disease or trypanosomiasis	
Suramina	Protozoans flagellates	Site of action present only in parasite cell
Albendazole	Helminthhs, microsporidia	Different targets within the parasite and in the host
Eflornithine	Protozoans flagellated (american trypanosomi-asis or Chagas disease)	
Antimonials pentavalent	Tissue protozoans : leishmaniasis, African trypanosomiasis	Greater toxic effect in the parasite than in the host
Melarsoprol		

Next, we display some antiparasitics and their pharmacological targets, which are indicated by Arabic numbers: 1. Proguanil, 2. Benznidazole, Nifurtimox, Chloroquine, Quinine, Metronidazole, 3. Amphotericin B, 4. Primaquine, 5. Chloroquine, 6. Albendazole, Mebendazole, triclabendazole, Praziquantel, Ivermectin (Fig. 1).

Secondary effects of antiparasitic drugs lead to incompletion of treatment by the choice of the patient, which aggravates the risk of propagation of resistant strains and the evasion of the host’s immune system due to the presence of variations on the surface proteins of the parasite. Therefore, the development of vaccines is an important challenge for the control of infections caused by parasites (24).

Research strategies for antiparasitics mainly consider the study of genes that codify enzymes involved in activation mechanisms of antiparasitic drugs, inhibition of important cellular functions, induction of programmed parasite death, study of the factors of virulence or pathogenicity, among others (26).

### Pharmacogenomics and antiparasitics

Pharmacogenomics is a concept mentioned in literature since 1997. Before this year, the term “pharmacogenetics” (coined by Friedrich Vogel in 1959) was of common use, but because the acceptance of the definition of terms has not been formally established yet, authors use the words indistinctly^27^.

A consensus to differentiate the connotations of both terms is that “pharmacogenomics” considers the polygenic effect over the safe and effective response towards drugs, as well as there adverse effects, where “pharmacogenetics” approaches a monogenic effect that influences in the variability of pharmacological metabolism (28).

**Fig. 2:**
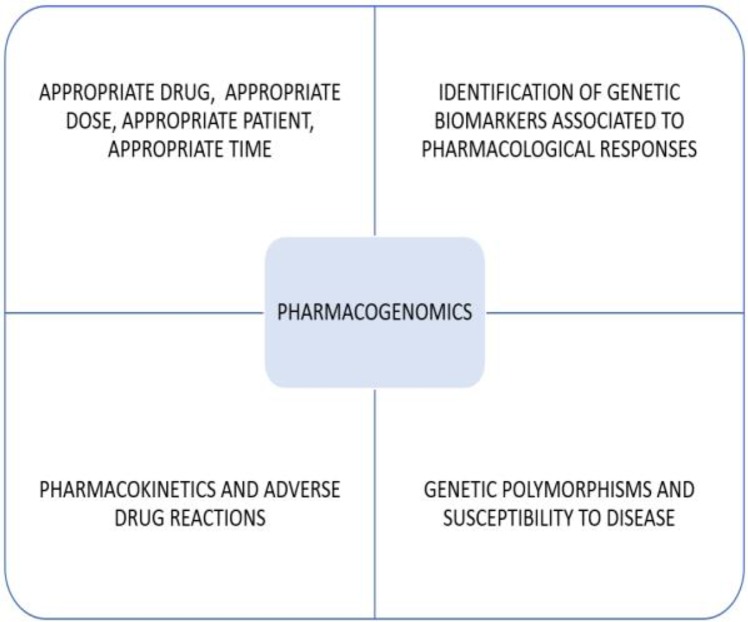
Field of application of pharmacogenomics in antiparasitics

Currently, pharmacogenetics is viewed as a branch of pharmacology that delves into genetic variability and its relation to the diverse interindividual and interpopulational responses towards drugs (29). The field of application of pharmacogenomics in drugs includes antiparasitics (Fig. 2).

Genetic variability, which distinguishes every single person, shows a difference as low as 0.1% in human genome; in other words, we share a genome with a similarity of 99.9% (30).

Genetic variability is established based on simultaneous existence in a population of genomes having distinct alleles for a particular locus. These changes are called “polymorphisms” and their presence in the human genome is approximately of 10––30 million (31).

The genetic difference, which distinguishes one human from other human beings, is expressed in various degrees, although not necessarily in a cause-effect scenario. Within the genetic variability among individuals and populations, there are phenotypical systems such as the ABO system, height, and eye color, to mention some. Other notable contrasts due to our genetic variability are the susceptibility to different diseases, as well as the diverse pharmacokinetic responses to drugs (30).

**Fig. 3:**
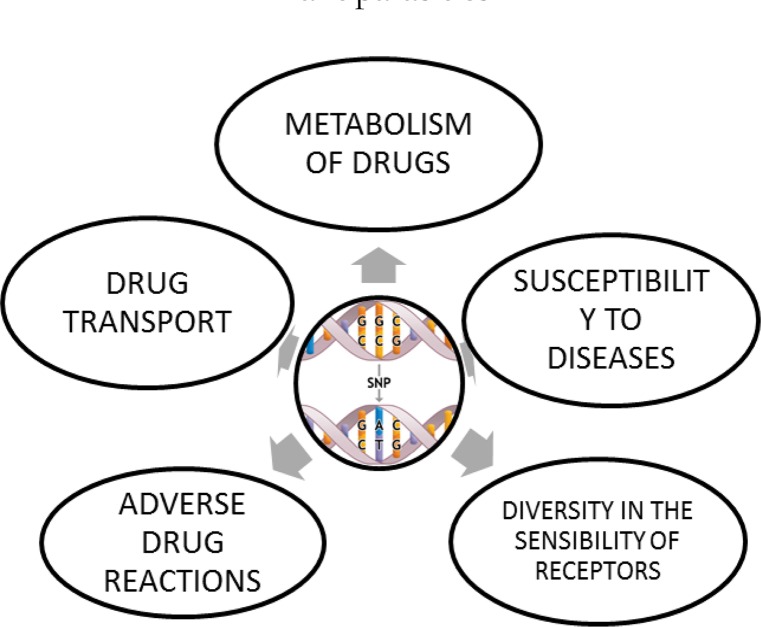
Effect of the genetic variability in the metabolism of antiparasitic drugs (41)

In this manner, the influence of variations in the genes that codify enzymes which correspondingly metabolize drugs leads to the appearance of adverse reactions after the administration of a drug, as well as in the rise of frequency in collateral effects, exacerbation of physiological reactions, resistance to treatment, susceptibility to diseases, alterations in drug metabolism, changes in drug transporters, modifications in receptors, among others (32) (Fig. 3).

A drug can be affected both in its pharmacokinetics and pharmacodynamics by the diverse genetic variations, and this is one of the factors that could explain why a given dose of a drug does not produce the desired effect in a group of patients, but instead it leads to toxicity in another and, in some cases, presents interactions with other drugs (32).

Only 25% to 60% of patients have an effective and safe response to the majority of drugs (33), including antiparasitics; the remaining percentage presents adverse drugs reactions (34). The WHO defines said reactions as “whatever response to a drug that is harmful, non-intended and that is produced at habitual doses for prophylaxis, diagnosis or treatment” (35).

The global notification program for adverse drug reactions was created in the 1960s by the WHO and accounts for 119 countries nowadays. Mexico began its participation in the program in 1999. The information is collected by pharmacies, the pharmaceutical industry, hospital pharmacovigilance units and state centers. It is provided through the National Center of Pharmacovigilance (CNFV) that depends directly from the Federal Commission for the Protection against Sanitary Risks (COFEPRIS) of the Secretary of Health, which reunites and directs the information to the surveillance center in Uppsala, Sweden, which is the operational coordinator of the drug surveillance program for the WHO (36–39).

Besides the genetics of the individuals, the interindividual variability in the response to drugs is multifactorial, comprising factors such as age, sex, ethnicity, anthropometry, lifestyle, diet, stress, drug interactions, previous or concomitant diseases.

Furthermore, drugs can provoke genome variations, such as the intermediary reduced hydroxylamine, which is a potential mutagen that is formed in the endogenic process involved in the reduction of metronidazole molecule, and for which the capability of generating punctual mutations has been proposed (40).

In the present day, the variations in around 20 genes provide useful predictions of reactions to drugs for at least 7% of drugs (80 to 100) approved by the FDA (34).

Around 500 genes in our genome are related to the response and safety of drugs. These genes can be studied from a pharmacological point of view in a differential manner, such as pharmacokinetic genes, which are those destined to codifying the proteins involved in the Absorption, Distribution, Metabolism and Elimination (ADME) process, among which enzymes of drug oxidative metabolism are found (isoforms of CYP), as well as plasmatic proteins and transporters, pharmacodynamic genes that implicate proteins, receptors, ion channels and immunomodulatory molecules (42, 43).

A deep study of the genes involved in the pharmacokinetics and pharmacodynamics of the antiparasitics will allow for the discovery of therapeutic targets that assist in the search for personalized treatment, these approach does not imply a prescription based treatment, but rather the selection or exclusion of patients that could present adverse drug reactions, for example patients showing poor, moderate or ultrarapid metabolisms. Pharmacogenomics allow to evaluating genetic alterations that give way to a diversity of responses to provide a more safe and effective drug, at the correct dose for the appropriate patient and with an optimal treatment duration, besides assisting in the development of vaccines (44).

### Study of the genetic variability in the pharmacological response

Whole Genome Association Studies (GWA) have demonstrated there is an association between single nucleotide polymorphisms (SNP) and parasitic disease. To identify and characterize the causal genes, linking and association studies have been employed (46).

Linking studies allow for the genotypification of microsatellites distributed throughout the whole genome, leading to the identification of genes presumably implicated in the physiology of the disease.

The most commonly used polymorphism to identify these genes is SNP. The “case-control” association studies are employed for the study of candidate genes, despite the following limitations (45, 46):

- It lacks replicability in different populations
- Small sample size
- Low statistical power
- Errors of genotypification
- Lack of precision in the clinical characterization of the disease
- Inadequate selection of the cases and controls
- Presence of populational sub-structure

### Markers of genetic variation towards antiparasitics

The biological variations related to the pharmacological response can appear in the genes that codify for the therapeutic targets, for the transporters and/or metabolizing enzymes. The genes, which codify enzymes related to the pharmacokinetics of the antiparasitics, are mainly related to the metabolism, transporters, plasmatic proteins and transcription factors.

The most common type of variations is SNP. Also variations in the gene of the human leucocitary antigen (HLA) and in the number of copies (CNVs) (47).

The variation in pharmacological response can be explained by the polymorphic variants in the codifying genes for:

- Metabolizing enzymes for cytochrome p450
- Transporters that allow the entry or facilitate the exit of drugs through the cell membrane
- Receptors
- Ion channels
- Plasmatic union proteins (albumin, acidic α-glycoprotein)
- Regulators and factors of transcription

Metabolism of antiparasitic drugs is mainly modified by variation in cytochrome p450 enzymes. These enzymes present a high frequency in polymorphic variability, which affects the expression of the isoforms CYP2C, CYP1A, CYP34A, CYP2D6, CYP2E1 and CYP2A6 (Table 4). The form of expression of the polymorphisms in the isoforms mentioned is related to those patients that metabolize poorly (5%–10%), intermediate metabolizers (10%–15%) or ultrarapid metabolizers (2%–7%).

**Table 4:** Interactions of induction or inhibition over cytochrome p450 (CYP) intestinal antiparasitic drugs commonly used in Mexico with substrates of the CYP administered with these drugs (55–58).

***Antiparasitic drugs***	***CYP Gene***											
	**1A1**	**1A2**	**2C8**	**2C9**	**2C19**	**2D6**	**3A4**	**3A5**	3A4*3	**3A7**	**19A**
Metronidazole			Inh	Inh			Inh				
				S			S				
Clotrimazole			Inh	Inh			Inh				
							Ind				
Tinidazole							S				Inh
Albendazole	Inh	Inh					S				
		S									
		Ind									
Tiabendazole	Ind	Inh									
		S									
Mebendazole	Ind						S				
Fenbendazole		Ind									
Pyrantel-oxantel						S					
Emetine						S	S				
						Ind	Ind				
Praziquantel		S			S	Inh	S	S	S	S	

Polymorphisms in the transporters can influence the codifying genes for transporters of ATP union (also called “ABC” transporters), genes for carrier transporters of solutes (SLC) or genes codifying for glycoprotein P (PgP), related to the efflux of xenobiotics and probable drug resistance.

In consequence, understanding the way in which antiparasitics interact with the pharmacological target facilitates the development of new and more efficient drugs. Thus we must consider the mechanism of action of the chemotherapeutic agent that relates to:

- Transporters (of influx and efflux)
- Metabolism (pharmacological potency)
- Pharmacological targets (enzymes, protein complexes, and metabolites)

With respect to transport of drugs, generally antiparasitics enter cells by passive transport mechanism, through membrane channels and transporters; a modification of these can modify drug specificity (48–55).

## Conclusion

Genetic variability contributes to interindividual diversity in the response to drugs. Enzymes that metabolize the majority of the antiparasitic drugs happened to be codified by genes that present genetic polymorphism. These polymorphic enzymes can cause alterations to the pharmacokinetics of the drug. In particular, they can alter the metabolism of antiparasitic drugs such as metronidazole, albendazole, mebendazole, emetine, praziquantel, which are among the most-used antiparasitic drugs in Mexico. The better understanding of the pharmacogenetics of antiparasitic drugs contributes to more accurate decision making in the selection of an antiparasitic drug, which lowers the risk of presentig adverse reactions. This is the search for personalized therapy.
